# Supplementary material for: Evaluation of the virtual learning environment by school students and their parents in Saudi Arabia during the COVID-19 pandemic after school closure
Source: PLoS One. 2022 Nov 2;17(11):e0275397. doi: 10.1371/journal.pone.0275397 (PMC9629622; doi:10.1371/journal.pone.0275397)
Supplement: S1 File — (PDF) [file pone.0275397.s001.pdf]

Evaluation of virtual learning environment during COVID-19 pandemic

Dear parents, we are group of researchers and Faculty members of King Abdulaziz University, Kingdom of Saudi Arabia. We are conducting a research project entitled: **"Assessment of online virtual learning environment during COVID-19 pandemic ".** This research aims to show the impact of the shifting to online teaching and assessment after school closure in the current pandemic crises on children and their families and provide solutions and/or recommendations to deal with this unexpected situation.

Therefore, as a mother, father or caregiver, you are invited to answer these questions
There are 8 questions in this survey.

General Questions

Your Nationality *

❗ Choose one of the following answers
Please choose **only one** of the following:

◯ Saudi
◯ Non-Saudi

Area of Living (Location) *

❗ Choose one of the following answers
Please choose **only one** of the following:

- ◯ Qassim Region
- ◯ Riyadh Region
- ◯ Tabuk Region
- ◯ Madinah Region
- ◯ Makkah Region
- ◯ Jawf Region
- ◯ Ha'il Region
- ◯ Al- Bahah Region
- ◯ Jizan Region
- ◯ Asir Region
- ◯ Najran Region
- ◯ Eastern Province

Your relationship with child *

❗ Choose one of the following answers
Please choose **only one** of the following:

- ◯ Father
- ◯ Mother
- ◯ Guardian

How many children do you have? *

❶ Choose one of the following answers

Please choose **only one** of the following:

- ◯ 1
- ◯ 2
- ◯ 3
- ◯ 4
- ◯ 5
- ◯ >5

Age, Gender and Grade of your eldest child *

	Age in Years	Gender of Child	Grade of Study
**Eldest Child**			

Parents Data *

	Age in Years	Education Level	Job Status
**Father**			
**Mother**			

Scale

Opinion of school children and their families in virtual learning environment (VLE)

If your child was going to school but used e-learning during the pandemic of novel Coronavirus, we need to record your response or answers for the older child only by answering the following questions for him or her.

The following answers relate to my eldest son or daughter

In a scale from 1-5, my child rates the following aspects of online teaching and assessment as *

Please choose the appropriate response for each item:

	Strongly agree 😍	Agree 😘	True sometimes 😐	Disagree 😟	Strongly disagree 😡
1-My child liked and enjoyed Virtual Learning Environment (VLE)	◯	◯	◯	◯	◯
2-My child found VLE easy	◯	◯	◯	◯	◯
3-My child found VLE useful	◯	◯	◯	◯	◯
4- My child found online VLE exciting and stimulating	◯	◯	◯	◯	◯
5- My child preferred online VLE over traditional education	◯	◯	◯	◯	◯
6- My child found that the instructor was interactive and facilitated discussions during online teaching session	◯	◯	◯	◯	◯
7- My child found that the instructor was accessible to answer questions or give feedback. during online teaching session	◯	◯	◯	◯	◯
8-My child found that the presentations of topics used during online sessions were clear and helpful	◯	◯	◯	◯	◯
9-My child was comfortable with participants from other students during online teaching session	◯	◯	◯	◯	◯
10- My child was comfortable and familiar with use of texting(writing) during online teaching session when needed	◯	◯	◯	◯	◯

	Strongly agree 😍	Agree 😘	True sometimes 😐	Disagree 😟	Strongly disagree 😡
11- My child was comfortable and familiar with use of Audio (microphone) during online teaching session when needed	◯	◯	◯	◯	◯
12- My child was comfortable and familiar with use of Video during online teaching session when needed	◯	◯	◯	◯	◯
13-My child had problems in availability of the required computer/ laptop/iPad/Tab	◯	◯	◯	◯	◯
14-My child found some difficulties in availability and connection with internet	◯	◯	◯	◯	◯
15-My child found online assessment and exams adequate	◯	◯	◯	◯	◯
16-My child missed interactive/Live learning (usual education)	◯	◯	◯	◯	◯
17-My child missed school activities	◯	◯	◯	◯	◯
18-My child missed gathering with friends	◯	◯	◯	◯	◯
19-My child missed gathering with teachers	◯	◯	◯	◯	◯
20-As a parent I'm satisfied with my role and efforts in helping my children during online teaching	◯	◯	◯	◯	◯
21-As a parent I find it easy task to help my children during their online teaching	◯	◯	◯	◯	◯
22-As a parent, I had a challenge to find time to help and support my child during online VLE	◯	◯	◯	◯	◯

	Strongly agree 😍	Agree 😘	True sometimes 😐	Disagree 😟	Strongly disagree 😡
**23-As a parent, I had a challenge to give IT help and technical support to my child during online VLE**	◯	◯	◯	◯	◯
**24-As a parent and overall, I considered myself successful in the management of my children's education and teaching.**	◯	◯	◯	◯	◯

At the end, you are welcome to add any further opinion or comment if you wish:

Submit your survey.
Thank you for completing this survey.
