# Supplementary material for: Evaluation of the virtual learning environment by school students and their parents in Saudi Arabia during the COVID-19 pandemic after school closure
Source: PLoS One. 2022 Nov 2;17(11):e0275397. doi: 10.1371/journal.pone.0275397 (PMC9629622; doi:10.1371/journal.pone.0275397)
Supplement: Attachment — Submitted filename: Response to Reviewers.docx [file pone.0275397.s003.docx]

**Dear Editor in Chief and Academic Editor of PLOS ONE Journal,**

Thank you for the revision of this original article (PONE-D-22-14889: Evaluation of the virtual learning environment by school students and their parents in Saudi Arabia during the COVID-19 pandemic after school closure) and giving us the chance to respond and explain some issues that can answer all concerns of editor/reviewer (s) and improve the written manuscript to meet PLOS ONE’s publication criteria.

Authors have responded to all comments and each point raised by the academic editor and reviewer (s) and all required necessary changes were added to the manuscript appropriately at the relevant sites highlighted in red color and within the requested time frame for revision.

A rebuttal letter that responds to each point raised by the academic editor and reviewer(s) was uploaded as a separate file labeled 'Response to Reviewers'. Both a marked-up copy of the manuscript with highlighted changes (Revised Manuscript with Track Changes) and an unmarked version of the revised paper without tracked changes labelled (Manuscript) were uploaded as 2 separate files.

**Reply to Comments of the Academic Editor:**

According to Plos One Requirements, authors would like to confirm that:

1. The manuscript meets PLOS ONE's style requirements, including those for file naming.

2. The additional details regarding participant consent were included in the Methods section.

3. A copy of the questionnaire or scale was included as a Supporting Information file.

4. The manuscript was edited for proper English language, grammar, punctuation, spelling, and overall style by one or more of the highly qualified native English speaking editors (The Editing certificate is provided as a Supporting Information file)

5. Accession number or DOIs necessary to access data of this survey study is provided.

6. Captions for Supporting Information files were included at the end of the manuscript.

7. The reference list was reviewed to ensure that it is complete and correct.

**Reply to Comments of Reviewer (1):**

1. The reviewer was concerned about the lack of information on the theoretical basis for the satisfaction construct, the theoretical foundations on which the questionnaire is based, and the definition of VLE and for satisfaction.

Response/Reply: Regarding definition of VLE, in the introduction section, VLE was defined as remote learners receive their learning materials electronically via the internet (Zhang, Zhao, Zhou, & Nunamaker, 2004). However, more details about particular VLE conducted in Saudi Arabia was added and highlighted in the methods section under the subheading (Study design and participant selection).

Regarding the theoretical foundation on which the questionnaire was based, it was mentioned in the methods section (subheading: Measuring tool) that the items of the questionnaire suitable for school students were based on previous studies and expert opinions from validated instruments for assessing distance education learning environments in higher education, integrative review for barriers and solutions to online learning and literature reviews and simultaneously included the opinion of parents in VLE ((Zhang, Zhao, Zhou, & Nunamaker, 2004 & Walker & Fraser, 2005 & Strong, Irby, Wynn & McClure, 2012 & O'Doherty et al., 2018).

In the methods section (Subheading: Statistical validation/reliability analysis of the questionnaire), it was mentioned that the used questionnaire was checked after its implementation by all possible statistical means and it was found to be of adequate and acceptable validity and reliability. Moreover, exploratory factor analysis was conducted for 24 variables (questions) using parallel analysis to determine the number of factors to retain with direct oblimin rotation. The following reference of another previous research applied the same method of statistical validation and reliability analysis and exploratory factor analysis used in our survey.

Hamutoglu NB, Gemikonakli O, Duman I. Kirksekiz A, Kiyici M. Evaluating students experiences using a virtual learning environment: satisfaction and preferences. Education Tech Research Dev. 2020; 68: 437-462.

According to exploratory factor analysis, the whole questionnaire consisted of 5 main domains:

- Learner domains (3): (perspective on the VLE, skill and interaction, perspective on traditional classrooms)

- Parent domains (2): (facilities/support and parental role/perspective on the VLE)

Regarding the definition for participant satisfaction about VLE, in the methods section (Subheading: Statistical validation/reliability analysis of the questionnaire), it was mentioned that the answers to the questions were rated on a 5-point Likert scale from 1-5 (strongly disagree=1, disagree=2, true sometimes=3, agree=4 and strongly agree=5).

In the methods section (Subheading: Questionnaire implementation and distribution), it was mentioned that in this study, learner and parental VLE satisfaction was the main dependent or outcome variable. A score with a median value above 3 indicated overall satisfaction with the VLE experience as well as satisfaction with each individual domain, while a median value below 3 indicated dissatisfaction. However, this important paragraph was transferred and highlighted under a new subheading Definition of the main outcome (VLE satisfaction) to be more prominent.

It is clear to choose “3” as an average score on five-point Likert scale. It is worth mentioning that we did not introduce any cut-off for satisfaction rather using “3” being the central category of the Likert scale to interpret the tendency of satisfaction scores.

The following reference adopted the same cutoff value and this reference was added to the reference list. The same value for measuring satisfaction to each question evaluating VLE was exactly the same value used in our survey The mean of all questions was calculated and all reported values above 3.0, the mid-point on the scale indicated satisfaction.

Barker J and Gossman P. The learning impact of a virtual learning environment: students' views. Teacher Education Advancement Network Journal (TEAN). 2013; 5:19-38.

**Reply to Comments of Reviewer (2):**

The reviewer asked if authors are talking about a particular VLE or about distance education at large, to add some details about the particular VLE in KSA, to know more about the pandemic in KSA (how many lockdowns? how long?) and to compare results with other researches made in other countries.

Response/Reply: More details about particular VLE conducted in KSA during COVID-19 crisis and relation of study time to number and time of lockdowns were added and highlighted in the methods section under the subheading (Study design and participant selection).

Regarding comparing results with other researches made in other countries. Authors thank the reviewer for giving the opportunity to emphasize that up to the best of our knowledge, this study is unique because it is the first comprehensive study either worldwide or from KSA to consider the opinion of both school students and their parents simultaneously (not in higher education setting as done in other previous researches) in the evaluation of satisfaction for different aspects of VLE.

However, it was mentioned in the discussion section that: To mitigate the spread of SARS-CoV-2 among children, considering that their safety is a priority, the governments of 188 countries introduced social distancing and lockdown measures with unprecedented worldwide closure of face-to-face child services such as schools interrupting education for >90% of the world’s students or 1.5 billion children and youth establishing a learning crisis (Human Rights Watch, 2020 & UNESCO b Education: From disruption to recovery, 2020 & Lee, 2020) While more than two-thirds of countries have introduced a national distance learning platform, only 30% of low-income countries have done so (UNESCO c, National learning platforms and tools, 2020 & Selbervik, 2020). The COVID-19 pandemic implies limited or no education for many children. Not only did school closure in many countries with planned extended lockdowns interrupted teaching but many exams have also been postponed, rescheduled or cancelled (Daniel, 2020 & UNESCO b, Education: From disruption to recovery, 2020).

Consequently, it was mentioned in the conclusion that: However, fortunately, the Saudi VLE experience is better than no education or the lack of facilities for any VLE for populations who are digitally-excluded without internet access in resource-poor countries.
